# Nuclei‐Specific Amygdala Enlargement Is Linked to Psychiatric Comorbidities in Drug‐Resistant Focal Epilepsy

**DOI:** 10.1002/acn3.70071

**Published:** 2025-05-19

**Authors:** Hélène Mourre, Julia Makhalova, Lisa Soncin, Elodie Garnier, Hugo Dary, Arnaud Le Troter, Roy A. M. Haast, Benoit Testud, Marie Arthuis, Samuel Medina Villalon, Stanislas Lagarde, Francesca Pizzo, Christian Bénar, Jean‐Philippe Ranjeva, Maxime Guye, Fabrice Bartolomei

**Affiliations:** ^1^ APHM, Timone Hospital Epileptology and Cerebral Rhythmology Marseille France; ^2^ Aix Marseille Univ, CNRS CRMBM Marseille France; ^3^ APHM, Timone Hospital CEMEREM Marseille France; ^4^ Aix Marseille Univ, INSERM, INS Inst Neurosci Syst Marseille France

**Keywords:** amygdala, epilepsy, psychiatric comorbidities

## Abstract

**Objective:**

Amygdala enlargement has been the subject of controversial studies regarding its significance in terms of pathogenicity both in epilepsy and in psychiatric comorbidities such as anxiety, depression, and post‐traumatic stress disorder. However, no causal link has been established in either direction, and the role of distinct amygdala nuclei remains unknown. We investigated volumetric changes of the amygdala and its nine main nuclei and their associations with psychiatric comorbidities in patients with drug‐resistant focal epilepsy.

**Methods:**

Eighty‐seven adult patients with drug‐resistant focal epilepsy, available 7 T MRI, and completed standardized psychiatric assessments were included. Whole amygdala and nuclei volumes were quantified and compared to healthy controls. Correlations between the amygdala or nuclei volumes and psychiatric scores were analyzed, as well as the prevalence and severity of each comorbidity depending on the presence of enlargement.

**Results:**

Amygdala enlargement was present in 41% of patients, with bilateral enlargement observed in 30% of these cases, while atrophy was noted in 2%. Bilateral enlargement correlated with higher posttraumatic stress disorder and depression scores. Central nucleus enlargement was associated with a greater prevalence of depression and more severe anxiety. Bilateral enlargement of distinct nuclei in the basolateral group was linked to more severe depression or posttraumatic stress disorder.

**Interpretation:**

These findings suggest that bilateral amygdala enlargement, particularly in specific nuclei, may serve as a morphological marker of psychiatric comorbidities in epilepsy. Further research is needed to explore the specific roles of amygdala nuclei in psycho‐epileptogenesis.

## 1 Introduction

Amygdala enlargement (AE) is a radiological abnormality increasingly reported in patients with epilepsy. However, its pathological significance is not elucidated. It was first described in temporal lobe epilepsy (TLE) in 1999, in association with dysthymia [1]. Subsequently, numerous studies have reported AE in different types of epilepsy [2, 3, 4, 5, 6, 7], in particular in TLE without identifiable lesions on imaging, reporting a prevalence of AE ranging from 12% to 63% [3, 4, 7]. Some authors have suggested that AE is an epileptogenic lesion similar to hippocampal sclerosis [5, 8]. However, recent data argue against the hypothesis of a distinct epileptic syndrome or lesion. Although the prevalence of AE is higher in patients with focal epilepsy than in those with idiopathic generalized epilepsy and healthy controls, it is not significantly higher in temporal than in extra‐temporal epilepsies [7]. Moreover, AE can be found outside the epileptogenic zone, and its localizing value appears limited, with the majority of AE being bilateral or contralateral to epilepsy in several studies [7, 9, 10]. In TLE, surgically resected enlarged amygdala disclosed normal histopathology in most cases [11].

The amygdala is characterized by strong functional and structural connectivity with the rhinal cortex and the hippocampus [12], and is involved in the genesis of temporal seizures [13]. The role of the amygdala in epileptogenesis has been established previously [14, 15]. Yet a recent study by our group has shown that in drug‐resistant focal epilepsy, the enlarged amygdala is heterogeneous in terms of epileptogenicity [10]. At the same time, the amygdala is particularly involved in emotional memory processing, encoding and extinction of fear memories, and triggering behaviors adapted to danger [16, 17, 18]. Independently of epilepsy, AE is described in multiple psychiatric disorders, in particular anxiety [19], depression [20] and post‐traumatic stress disorder (PTSD) [21]. However, amygdala volumetric data in this context are inconsistent across studies, with reports of enlargement [20, 21, 22] and atrophy [23, 24]. Furthermore, several amygdala nuclei have been suggested as contributing to the observed functional and morphological alterations [22, 25, 26]. However, establishing clear anatomo‐functional correlations is hampered by the lack of precise assessment of the amygdala and its various nuclei that are challenging to delimit at conventional magnetic field strengths. Therefore, there is a need for a robust, automated volumetry approach based on ultra‐high field 7 T MRI [27].

Psychiatric disorders are significantly more common in patients with epilepsy compared to the general population. For instance, the prevalence of anxiety is estimated to be 30% in patients with epilepsy, compared to 9% in the general population. Similarly, depression affects 31% of patients with epilepsy versus 10.7% in the general population, and PTSD occurs in 26% of patients with epilepsy compared to 7% in the general population [28, 29]. The links between epileptogenic and psychogenic processes seem complex. Some animal models suggest that psycho‐traumatism and repeated stress can induce epilepsy [30]. Conversely, persistent seizures contribute to the patient's psychopathological state [31]. The amygdala volume changes could reflect these complex interactions.

In the present work, we investigated volumetric changes of the whole amygdala and its nine principal nuclei, as measured using 7 T MRI, and their links with psychiatric disorders in patients with drug‐resistant focal epilepsy.

## 2 Methods

### 2.1 Patients and Clinical Data

**FIGURE 1:**
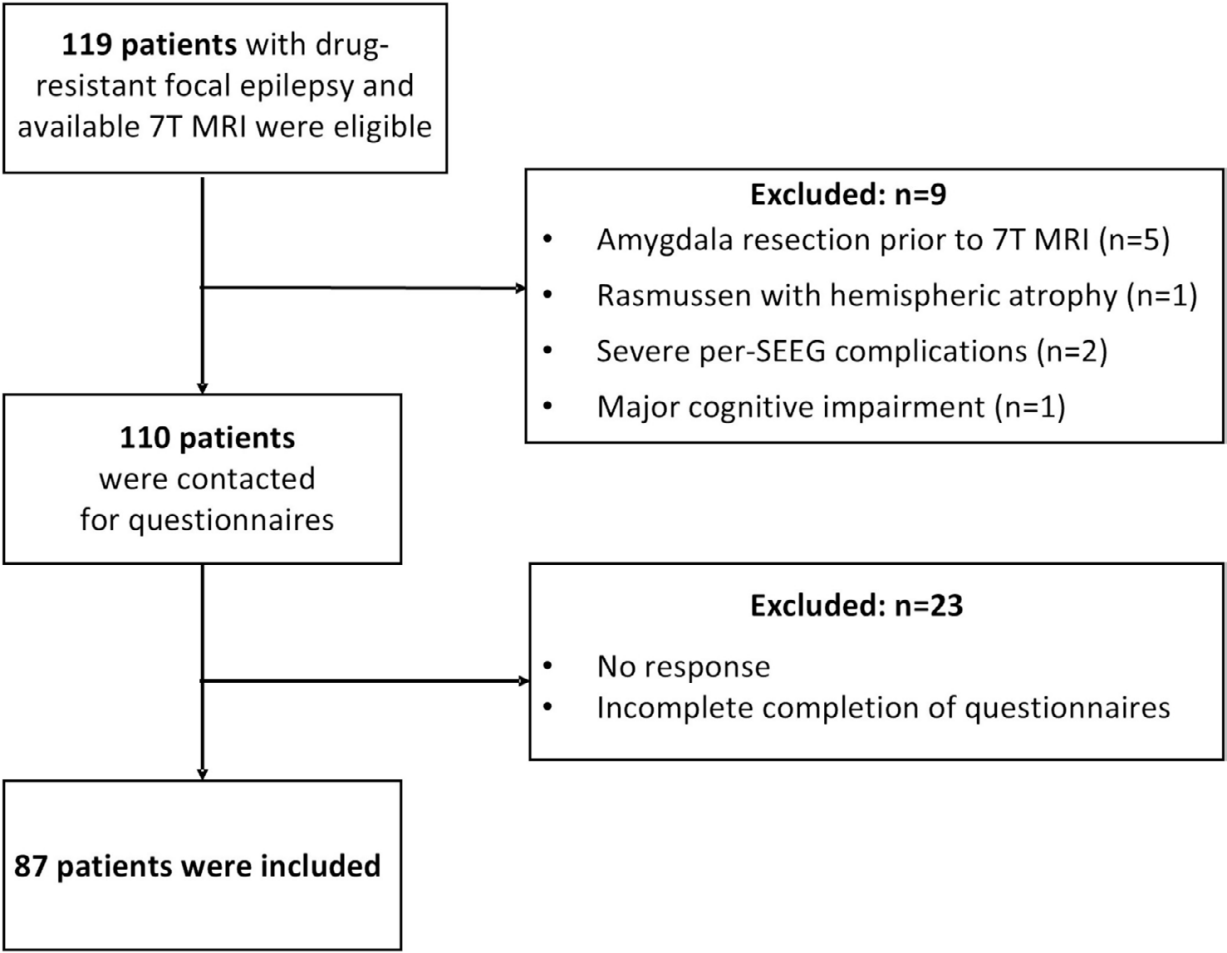
Flow‐chart of patients' inclusion.

All consecutive adult patients with drug‐resistant focal epilepsy who underwent 7 T MRI during the pre‐surgical assessment of their epilepsy at the Timone Epileptology Center in Marseille were eligible. Brain imaging data was acquired through two prospective studies: RHU EPINOV (NCT03643016) and NEURO7T (2016‐A01785‐46). The pre‐surgical workup included a detailed history, clinical and neuropsychological assessments, 3 T brain MRI, ^18‐^fluorodeoxyglucose (FDG)‐PET, video‐EEG recording in all patients, and stereo‐electro‐encephalography (SEEG) in a subset of patients. The inclusion steps are summarized in the flowchart (Figure 1). Of the 119 eligible patients, we excluded five with amygdala resection before 7 T MRI, one with hemispheric atrophy related to Rasmussen's encephalitis, two with severe pre‐SEEG complications, whose subsequent psychological trauma would constitute a significant bias, and one whose cognitive impairment compromised psychiatric evaluation. The remaining patients were contacted to complete psychiatric questionnaires (see below). The final cohort included 87 patients for whom imaging data and psychiatric scores were available. All subjects gave their informed written consent to participate in the study. The study was approved by Assistance Publique—Hôpitaux de Marseille (registration number health data access portal PADS VCRFY5).

Patient data included demographics, epilepsy onset age, duration, lateralization, type, clinical hypothesis of the epileptogenic zone topography, seizure frequency, psychiatric assessment age, imaging findings, SEEG/surgery results, histopathology, and seizure outcomes (Engel class). Epilepsy types were classified by the epileptogenic zone topography (temporal, frontal, central‐premotor, insulo‐opercular, posterior, multilobar), and etiologies were grouped as focal cortical dysplasia, other structural causes, or unknown.

### 2.2 Psychiatric Comorbidity Scales

The French version of the Neurological Disorders Depression Inventory‐Epilepsy (NDDI‐E) [32], a screening scale for major depressive disorder, was used, with a cut‐off score of 15/24. To screen for generalized anxiety disorder, we used the French version of the 7‐item Generalized Anxiety Disorder Scale (GAD‐7) [33], with a cut‐off score of 7/21. To screen for post‐traumatic stress disorder, we used The Posttraumatic Stress Disorder Checklist for Diagnostic and Statistical Manual of Mental Disorders‐5 (PCL‐5), a psychometric scale validated in France [34], with a cut‐off score of 31/80. Questionnaires were conducted blinded to the results of the MRI. Responses to the scale questionnaires were collected by telephone after written instructions had been sent by email (HM, LS).

All included patients underwent a systematic psychiatric evaluation using NDDI‐E and GAD‐7 prior to this study as part of their presurgical work‐up, according to the routine practice of our center. In 31 patients who participated in the RHU EPINOV (NCT03643016) study, this evaluation also included the French‐translated versions of the Beck Depression Inventory (BDI) and the State–Trait Anxiety Inventory (STAI state and STAI trait). Fifty‐six patients had a detailed evaluation by a psychiatrist (MA), including the Mini International Neuropsychiatric Interview (MINI).

### 2.3 MRI Acquisition and Visual Assessment

Ultra‐high‐field 7 T MR images were acquired in all patients and in 32 healthy controls (HC) on a 7 T MAGNETOM or MAGNETOM Terra MR system (Siemens Healthineers, Erlangen, Germany) using a 32‐channel (32Rx/1Tx) head coil. The protocol included a 3D T_1_‐weighted magnetization‐prepared 2 rapid acquisition gradient echoes sequence (T_1_w MP2RAGE, TR = 5000 ms/TE = 3 ms/TI1 = 900 ms/TI2 = 2750 ms, 256 slices, spatial resolution = (0.6 × 0.6 × 0.6) mm^3^, acquisition time = 10 min) for high‐resolution anatomical imaging. The visual quality check of 7 T MP2RAGE images was performed (JM, HD, BT) in all patients and HC, and the subjects with artifacts or resection cavities in the mesial temporal regions were excluded.

### 2.4 Volumetric Analysis of the Amygdala and Its Nuclei on 7 T MRI

**FIGURE 2:**
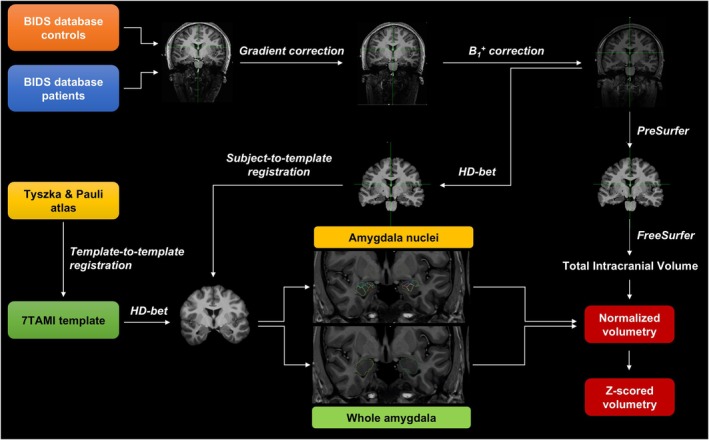
Pipeline for automated, atlas‐based volumetric segmentation of the amygdala and its nuclei using 7 T MRI template. Automated volumetric segmentation of the amygdala and its nine nuclei was performed in all patients and healthy controls (*n* = 32), using a combination of two atlases: The 7TAMI atlas for the whole amygdala and the Tyszka and Pauli atlas for the amygdala nuclei. The segmentation was performed using 7 T MP2RAGE images on high‐resolution 7TAMI template. Pre‐processing included correcting for gradient distortion, B1+ inhomogeneity distortion and creating a denoised image, followed by non‐linear registration of the 7TAMI model to individual anatomy. Nine different nuclei were segmented for each amygdala: AAA, anterior amygdaloid area; ASTA, amygdalo‐striatal transition area; ATA, periamygdaloid cortex; BL, basolateral; BM, accessory basal; CEN, central nucleus; CMN, corticomedial; LA, lateral; PL, paralaminar. Volumes were normalized using intracranial volume measurements, and *z*‐scores were calculated by comparing patient amygdala and nuclei volumes to control subjects' volumetry.

The volumetry pipeline is illustrated in Figure 2 and described in Data S1. In brief, automated volumetric segmentation of the amygdala and its nuclei was performed in all patients and HCs, combining the segmentation of the whole amygdala based on the in‐house developed 7TAMI atlas [10] with the segmentation of the amygdala nuclei based on the atlas by Tyszka and Pauli [27] on the 7TAMI template [35]. Firstly, the Tyszka and Pauli template was registered to the high‐resolution 7TAMI template [35] and the transformation was applied to the Tyszka and Pauli atlas. For all subjects, pre‐processing steps were performed on MP2RAGE to prepare the data. Segmentation was obtained by non‐linear registration of the 7TAMI model with the skull‐stripped UniDen (T_1_weighted) image that was then applied to the whole amygdala segmentation (7TAMI) and the nuclei atlas (Tyszka and Pauli). Nine nuclei were segmented for each amygdala: Lateral nucleus (LA); Basolateral nucleus (BL); Accessory basal nucleus (BM); Paralaminar nucleus (PL); Corticomedial nucleus (CMN); Periamygdaloid cortex (ATA); Anterior amygdaloid area (AAA); Amygdalo‐striatal transition area (ASTA); Central nucleus (CEN). Volumetric measurements were extracted from left and right amygdala segmentation masks adapted to the subject's anatomy for each patient and healthy control. The left and right amygdala volumes were normalized by the total intracranial volume.

The *z*‐scores of normalized amygdala volumes were then calculated for each patient compared with the volumetry of control subjects for the whole amygdala and the nine nuclei. In this study, the presence of amygdala enlargement was defined by a *z*‐score ≥ 1.5 to include cases with a moderate enlargement (1.5 ≤ zscore < 2) [2, 10]. Significant enlargement was determined by a *z*‐score ≥ 2. Statistical analyses were performed for both thresholds (*z* ≥ 1.5 and *z* ≥ 2).

### 2.5 Statistical Analysis

Statistical analyses were performed using R software (version 4.0.4) [36]. Psychiatric comorbidities were analyzed as continuous variables, using the NDDI‐E, GAD‐7, and PCL‐5 scores, and as categorical variables, based on the psychiatric score above the cut‐off for a high probability of depression (yes/no), anxiety (yes/no), and PTSD (yes/no). Similarly, the amygdala and nuclei volumes were analyzed as continuous variables using the *z*‐score values, and as categorical variables based on the presence of AE (yes/no) according to the thresholds *z* ≥ 1.5 and *z* ≥ 2. The analyses were performed for the whole amygdala and for each of the nine sub‐nuclei. Pearson correlation tests were used to study relationships between two continuous variables: (i) inter‐psychiatric score correlations (NDDIE, GAD‐7, and PCL‐5); (ii) correlations between the amygdala or nuclei volume *z*‐scores and the psychiatric scores in the whole cohort, as well as in the sub‐cohorts with and without AE. In patients with AE, these correlations were analyzed depending on the location of AE according to the epilepsy side (ipsilateral, contralateral, or ipsi/contralateral, the latter corresponding to a bilateral AE), epilepsy lateralization (left, right, bilateral), and epilepsy type (temporal, extra‐temporal). The Chi‐Square test was used to identify differences in the proportion of patients above the cut‐off scores for a high probability of depression, anxiety, or PTSD between patients with and without amygdala enlargement, as well as between patients with ipsilateral, contralateral, or bilateral AE. ANOVA followed by posthoc Student's test for statistically significant factors was used to assess relationships between a continuous variable and one or more categorical variables. In this way, we assessed group differences in psychiatric scores between patients with and without AE. Further, in the sub‐cohort of patients with AE, we investigated group differences in psychiatric scores depending on the AE location according to the epilepsy side: ipsilateral, contralateral, or ipsi/contralateral. Finally, the relationships between amygdala volume *z*‐scores and clinical cofactors (age at epilepsy onset, duration of epilepsy, seizure frequency, presence of MRI‐visible lesion, etiology) were assessed using the Pearson test for continuous variables and Student's *t*‐test for categorical variables. Multivariate analysis using complex models was performed to control for confounding factors such as seizure frequency, MRI‐visible lesion, epilepsy type, and etiology.

The Benjamini‐Hochberg false discovery rate (FDR) control procedure was used to adjust for multiple comparisons, with the threshold set at < 0.05 for statistical significance (*p* < 0.05). FDR correction was applied separately within each family and subgroup of analyses specified above.

## 3 Results

### 3.1 Clinical Characteristics of the Patient Cohort

The clinical characteristics of the 87 included patients are summarized in Table 1. The mean age at evaluation was 34.8 ± 10.5 years, the age at epilepsy onset was 16.4 ± 10.7 years, and the mean duration of the disease was 18.3 ± 10.5 years. Regarding the types of epilepsy, 58.6% were temporal (*n* = 51), 41.4% extra‐temporal (*n* = 36), of which 12.6% were insulo‐opercular (*n* = 11), 10.3% frontal (*n* = 9), 10.3% central‐premotor (*n* = 9), 5.7% multilobar (*n* = 5), and 2.3% posterior (*n* = 2). Epilepsy was left‐sided in half of cases (50.6%, *n* = 44), right‐sided in 40.2% (*n* = 35), and bilateral in 9.2% (*n* = 8). An MRI‐visible lesion was present in 45 patients (51.7%), while 42 patients (48.3%) had normal imaging. 59.8% of patients underwent SEEG exploration (*n* = 52), and 39.1% (*n* = 34) were operated on, with complete seizure freedom achieved in 58.8% (*n* = 20, Engel class I). Based on MRI and histological data, we identified 24 focal cortical dysplasias, eight neurodevelopmental tumors, seven polymicrogyria/heterotopias, five cases of gliosis, three vascular malformations, two hypothalamic hamartomas, and two cases of hippocampal sclerosis. In the remaining 36 patients, no lesions were identified.

**TABLE 1:** Clinical characteristics of the patients.

Epidemiology	
Sex, male/female, % (*n*)	54.0% (47)/46.0% (40)
Age at evaluation, years, mean ± SD (range)	34.8 ± 10.5 (19–61)
Age at epilepsy onset, years, mean ± SD (range)	16.4 ± 10.7 (0–40)
Epilepsy duration, years, mean ± SD (range)	18.3 ± 10.5 (3–47)
Seizure frequency, per month, mean ± SD (range)	31.7 ± 73.5 (1–600)
Epilepsy type	
Epilepsy Side, left/right/bilateral	50.6% (44)/40.2% (35)/9.2% (8)
TLE/NTLE	58.6% (51)/41.4% (36)
Epileptogenic zone (EZ) localization	
Temporal, % (*n*)	58.6% (51)
Frontal, % (*n*)	10.3% (9)
Insulo‐opercular, % (*n*)	12.6% (11)
Posterior, % (*n*)	2.3% (2)
Multilobar, % (*n*)	5.7% (5)
Central‐premotor, % (*n*)	10.3% (9)
SEEG, yes/no, % (*n*)	59.8% (52)/40.2% (35)
Surgery, yes/no, % (*n*)	39.1% (34)/60.9% (53)
Outcome (Engel class)	
I, % (*n*)	58.8% (20)
II, % (*n*)	17.6% (6)
III, % (*n*)	5.9% (2)
IV, % (*n*)	17.6% (6)
MRI and etiology	
MRI, normal/lesion, % (*n*)	48.3% (42)/51.7% (45)
Etiology, unknwown/structural, % (*n*)	43.7% (38)/56.3% (49)
Lesion type (MRI and histological findings if available), *n*	
FCD	24
Polymicrogyria/Heterotopia	7
DNET/Ganglioglioma/other low‐grade tumor	8
Hippocampal sclerosis	2
Hypothalamic Hamartoma	2
Vascular malformation	3
Gliosis	5
No lesion	36
Psychiatric comorbidities	
Anxiety, yes/no	86.2% (75)/13.8% (12)
Depression, yes/no	37.9% (33)/62.1% (54)
PTSD, yes/no	27.6% (24)/72.4% (63)

*Note:* Data are presented as mean ± SD (range) or % (*n*).

Abbreviations: DNET, dysembrioplastic neuroepithelial tumor; FCD, focal cortical dysplasia; HS, hippocampal sclerosis; MRI, magnetic resonance imaging; NTLE, no temporal lobe epilepsy; SEEG, StereoElectro‐Encephalography; TLE, temporal lobe epilepsy.

### 3.2 Psychiatric Scales Scores

86.2% of patients (*n* = 75) had scores above the cutoff for GAD‐7, 37.9% (*n* = 33) of patients had scores above the cutoff for NDDI‐E, and 27.6% of patients (*n* = 24) had scores above the cutoff for PCL‐5.

The personal history was positive for at least one psychiatric comorbidity in 57 (65.5%) out of 87 included patients. All patients who screened positive for a high probability of depression (NDDI‐E > 15, *n* = 33) or PTSD (PCL‐5 > 31, *n* = 24) had a history of the respective psychiatric comorbidity, with the diagnosis confirmed by a psychiatric evaluation including a structured interview (MINI) performed by a psychiatrist (MA). Both conditions co‐occurred with other comorbidities: depression with anxiety in 16 patients, depression with PTSD in one patient, PTSD with anxiety in seven patients, and depression with both PTSD and anxiety in 16 patients. Among the 75 patients who met the screening diagnostic criteria for a high probability of generalized anxiety disorder (GAD‐7 > 7), 39 had a history of anxiety associated with comorbid depression, PTSD, or both, as described above. Seventeen patients had a past history of isolated anxiety, established through a clinical interview by a psychiatrist (MA) and/or a positive screening using GAD‐7 and STAI.

Exposure to a traumatic event was reported in 55 patients (63%). The mean values above the cut‐off for anxiety scores were 12.6 ± 3.1 (GAD‐7), for depression 18.0 ± 2.0 (NDDI‐E), and for PTSD 41.1 ± 7.3 (PCL‐5), respectively. The assessed comorbidities were highly interrelated, with strong positive correlations between PCL‐5 and NDDI‐E (*r* = 0.65, *p* < 0.001), NDDI‐E and GAD‐7 (*r* = 0.61, *p* < 0.001), and GAD‐7 and PCL‐5 (*r* = 0.45, *p* < 0.001).

### 3.3 Amygdala Volumetric Characteristics

#### 3.3.1 Whole Amygdala Volumes

**FIGURE 3:**
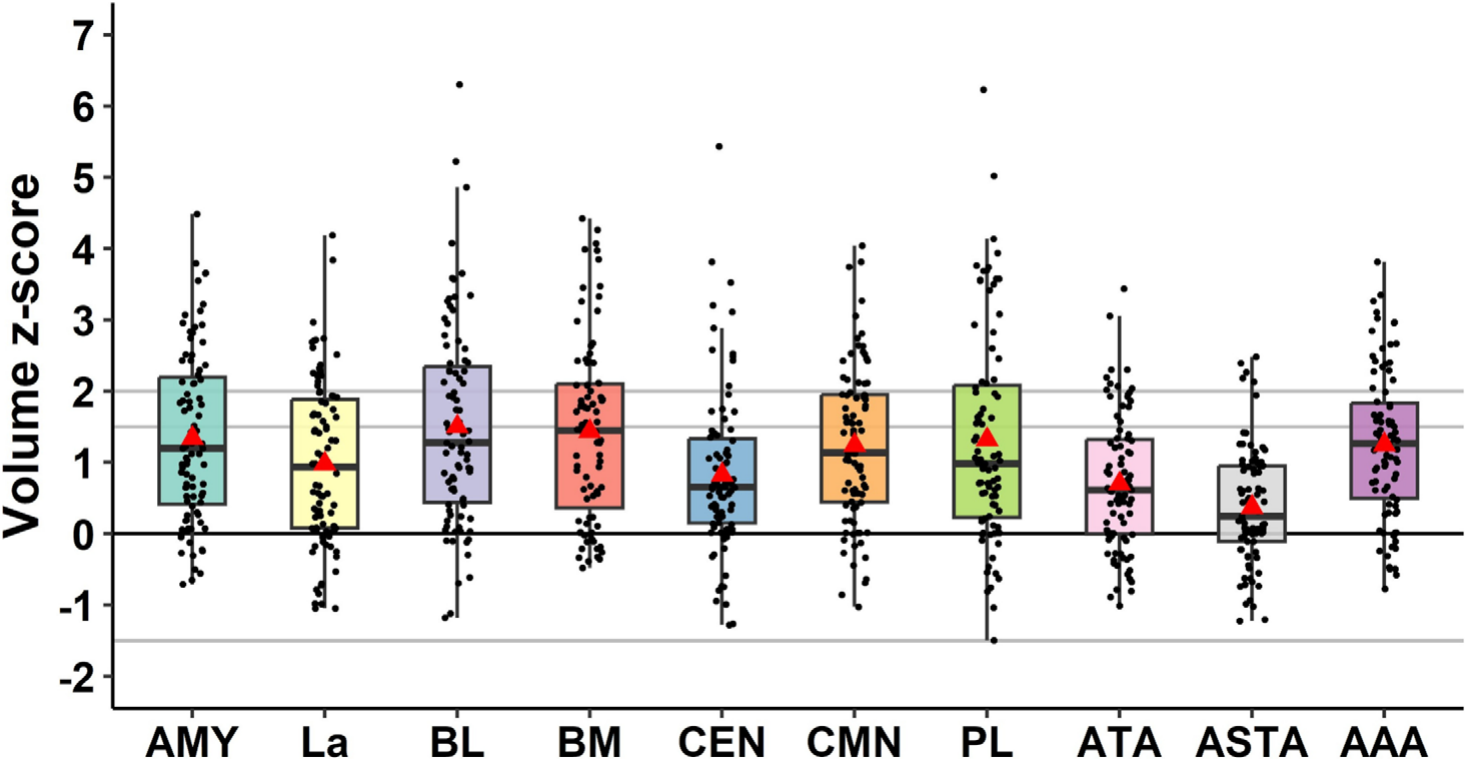
Amygdala nuclei volumes in the cohort of 87 patients with drug‐resistant focal epilepsy. The maximal normalized total amygdala and nuclei volume per patient (*z*‐scores vs. healthy controls) are shown. For each structure, the mean *z*‐scores are indicated by the red triangles and the medians by the black lines. The gray lines show the cut‐off *z*‐score values for a moderate (> 1.5) and significant (≥ 2.0) amygdala or nuclei enlargement, or an atrophy (≤ 1.5), respectively. AAA, anterior amygdaloid area; AMY, amygdala; ASTA, amygdalo‐striatal transition area; ATA, periamygdaloid cortex; BL, basolateral nucleus; BM, accessory basal nucleus; CEN, central nucleus; CMB, corticomedial nucleus; La, lateral nucleus; PL, paralaminar nucleus.

Volumetric data are detailed in Table 2 and Figure 3. AE (*z*‐score ≥ 1.5) was present in 41.4% of cases (*n* = 36) and detected more frequently contralateral (17.2%, *n* = 15) than bilateral (12.6%, *n* = 11) or ipsilateral to the epilepsy side (11.4%, *n* = 10). Among the patients with AE, 72.2% (*n* = 26) had a significant enlargement (*z*‐score ≥ 2), representing 29.9% of the total cohort. In this subgroup, the percentages of contralateral, bilateral, and ipsilateral enlargement were 12.6% (*n* = 11), 11.5% (*n* = 10), and 5.8% (*n* = 5), respectively. Only two patients (2.3%) had amygdala atrophy (*z*‐score ≤ −1.5), one of which was ipsilateral and another contralateral to the epilepsy side. The prevalence of AE was similar in patients with normal MRI (40.5%, *n* = 17) and those with lesional MRI (35.2%, *n* = 19), as well as in temporal (37.3%, *n* = 19) and extra‐temporal epilepsies (47.2%, *n* = 17). In the subgroup of operated patients, the percentage of seizure‐freedom (Engel class I) did not differ between the groups with AE (58.3%, *n* = 7) and without AE (59.0%, *n* = 13).

**TABLE 2:** Amygdala and nuclei volumetry results.

Whole amygdala	
Amygdala enlargement (*z*‐score ≥ 1.5), % (*n*)	41.4% (36)
Location to epilepsy side	
Ipsilateral	11.4% (10)
Contralateral	17.2% (15)
Bilateral	12.6% (11)
Significant AE (*z*‐score ≥ 2), % (*n*)	29.9% (26)
Location to epilepsy side	
Ipsilateral	5.75% (5)
Contralateral	11.5% (10)
Bilateral	12.6% (11)
Amygdala atrophy (*z*‐score < −1.5), % (*n*)	2.3% (2)

Amygdala nuclei		Location to epilepsy side (*n*)
	Total	Ipsi/contra/bilateral
Nuclei enlargement (*z*‐score ≥ 1.5), % (*n*)		
Lateral (La)	36.8% (32)	12.6% (11)/12.6% (11)/11.5% (10)
Basolateral (BL)	42.5% (37)	12.6 (11)/11.5% (10)/18.4% (16)
Accessory basal (BM)	47.1% (41)	17.2% (15)/14.9% (13)/14.9% (13)
Paralaminar (PL)	37.9 (33)	12.6% (11)/10.3% (9)/14.9% (13)
Central (CEN)	18.4% (16)	5.7% (5)/4.6% (4)/8.0% (7)
Corticomedial (CMN)	42.5% (37)	12.6% (11)/12.6% (11)/17.2% (15)
Periamygdaloid Cortex (ATA)	20.7% (18)	9.2% (8)/5.7% (5)/5.7% (5)
Anterior Amygdaloid Area (AAA)	39.1% (34)	14.9% (13)/14.9% (13)/9.2% (8)
Amygdalostriatal Transition Area (ASTA)	6.9% (6)	4.6% (4)/1.1% (1)/1.1% (1)
Nuclei atrophy (*z*‐score < −1.5), % (*n*)		
Lateral (La)	3.4% (3)	1/2/0
Basolateral (BL)	4.6% (4)	2/2/0
Accessory basal (BM)	0	NA
Paralaminar (PL)	6.9% (6)	2/4/0
Central (CEN)	2.3% (2)	2/0/0
Corticomedial (CMN)	0	NA
Periamygdaloid Cortex (ATA)	5.7% (5)	2/3/0
Anterior Amygdaloid Area (AAA)	0	NA
Amygdalostriatal Transition Area (ASTA)	1.1% (1)	0/1/0

*Note:* Data are presented as % (*n*).

#### 3.3.2 Amygdala Nuclei Volumes

The volumes of the nine amygdala nuclei—lateral (La), basolateral (BL), accessory basal (BM), paralaminar (PL), central (CEN), corticomedial (CMN), peramygdaloid cortex (ATA), amygdalo‐striatal transition area (ASTA), anterior amygdaloid area (AAA)—were quantified. The results are summarized in Table 2 and Figure 3. An enlargement of different nuclei could be observed involving, in order of frequency, BM (47.1%), BL and CMN (42.5%), AAA (39.1%), PL (37.9%), La (36.8%), ATA (20.7%), CEN (18.4%), ASTA (6.9%). Thus, it mainly concerned the basolateral nuclear group (La, BL, BM, PL) and the corticomedial nucleus (Figure 3). The location of nuclei enlargement according to the side of epilepsy (ipsilateral, contralateral, or bilateral) is detailed in Table 2. While enlargement of the majority of nuclei was observed equally across ipsilateral, bilateral, or contralateral to the epilepsy side, enlargement of the BL and CMN tended to be more frequently observed bilaterally, while ASTA was enlarged more frequently ipsilaterally to the epilepsy side. Similar to whole amygdala atrophy, nuclear atrophy was rarely observed for the individual nuclei and involved, in order of frequency, PL (6.9%), ATA (5.7%), BL (4.6%), LA (3.4%), CEN (2.3%), and ASTA (1.1%). No atrophy was found for CMN, BM, and AAA. It could be ipsilateral or contralateral to the epilepsy side; there were no cases of bilateral atrophy.

### 3.4 Links Between the Amygdala Volume and Psychiatric Scales Scores

#### 3.4.1 Whole Amygdala Volume and Psychiatric Scores

**FIGURE 4:**
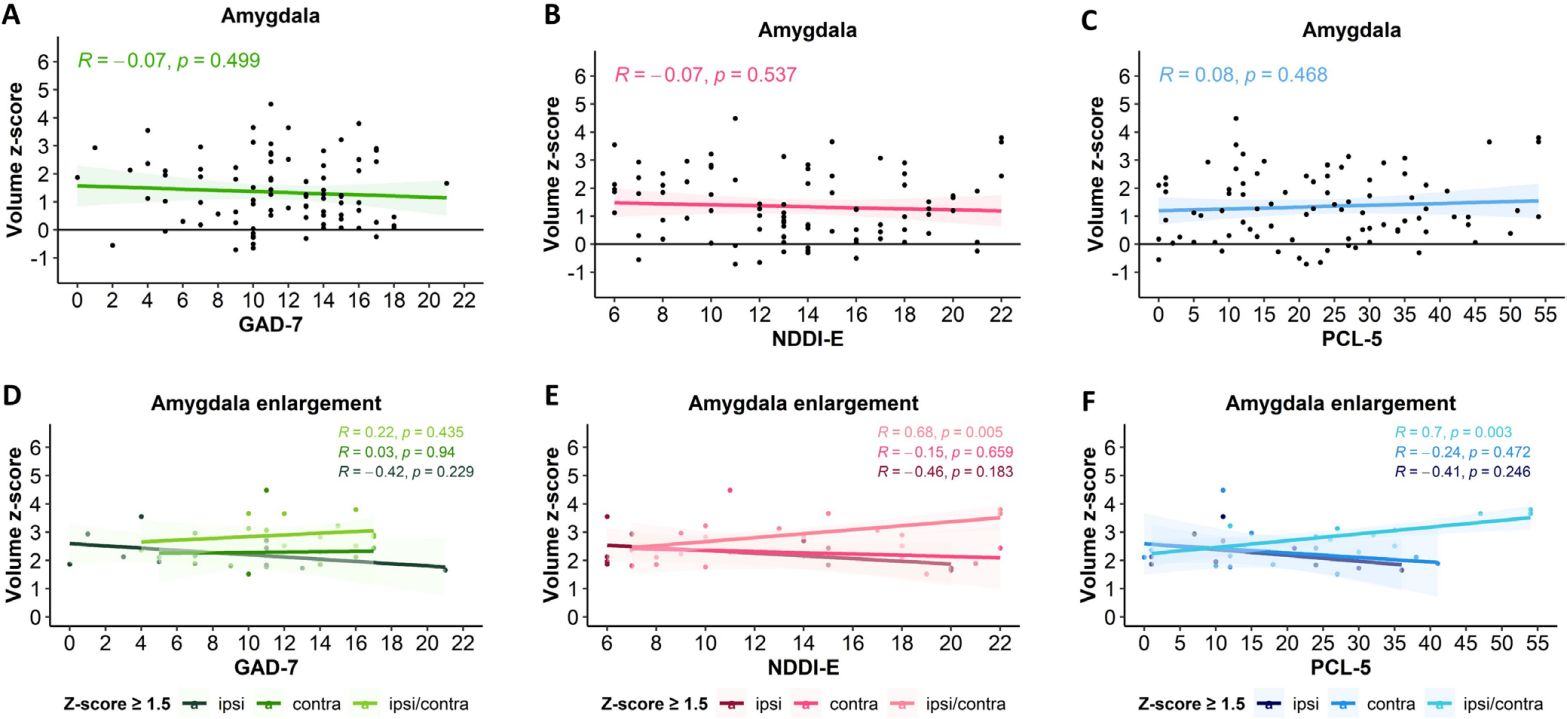
Correlations between the amygdala volume and the psychiatric scores. In the whole cohort (A–C), there was no correlation between the amygdala volume and the level of anxiety (A, GAD‐7), depression (B, NDDI‐E), or PTSD (C, PCL‐5) defined by clinical scores. Likewise, no correlation was found in the subgroup of patients without amygdala enlargement (not shown). In the subset of patients with amygdala enlargement (AE, D–F), no correlation was found between the amygdala volume and the level of anxiety (D). However, the amygdala volume positively correlated with the level of depression (E), as well as of PTSD (F), when bilateral AE (ipsi/contra) was present, while there was no correlation in cases of unilateral AE, whether ipsi‐ or contralateral to the epilepsy site.

In the whole patient cohort, no significant correlations were identified between amygdala volume and psychiatric scores for anxiety (GAD‐7), depression (NDDI‐E), or PTSD (PCL‐5) (Figure 4A–C). Furthermore, no differences were observed in the proportion of patients screened positive for a high probability of psychiatric comorbidities, nor in the mean psychiatric scores, between patients with and without AE. Likewise, no significant correlations between the amygdala volumes and psychiatric scores were found in the subgroup of patients without AE.

We then focused on the subgroup of patients with AE. Within this subgroup, no correlation was observed between amygdala volume and anxiety scores (GAD‐7). However, significant positive correlations were noted between the amygdala volume and depression (NDDI‐E, *p* = 0.005) and PTSD (PCL‐5, *p* = 0.003, Pearson) scores when bilateral AE (ipsi/contra) was present (both for *z*‐score ≥ 1.5 and *z*‐score ≥ 2.0). No such correlations existed for unilateral AE, whether ipsi‐ or contralateral to the side of epilepsy (Figure 4D–F). Furthermore, amygdala volume did not correlate with psychiatric scores depending on epilepsy lateralization (left, right, bilateral) or epilepsy type (temporal, extra‐temporal, Figure S1).

The proportions of patients screened positive for a high probability of anxiety, depression, and PTSD did not differ according to the ipsilateral, contralateral, or bilateral AE location with respect to the epilepsy side (Chi‐Square).

**FIGURE 5:**
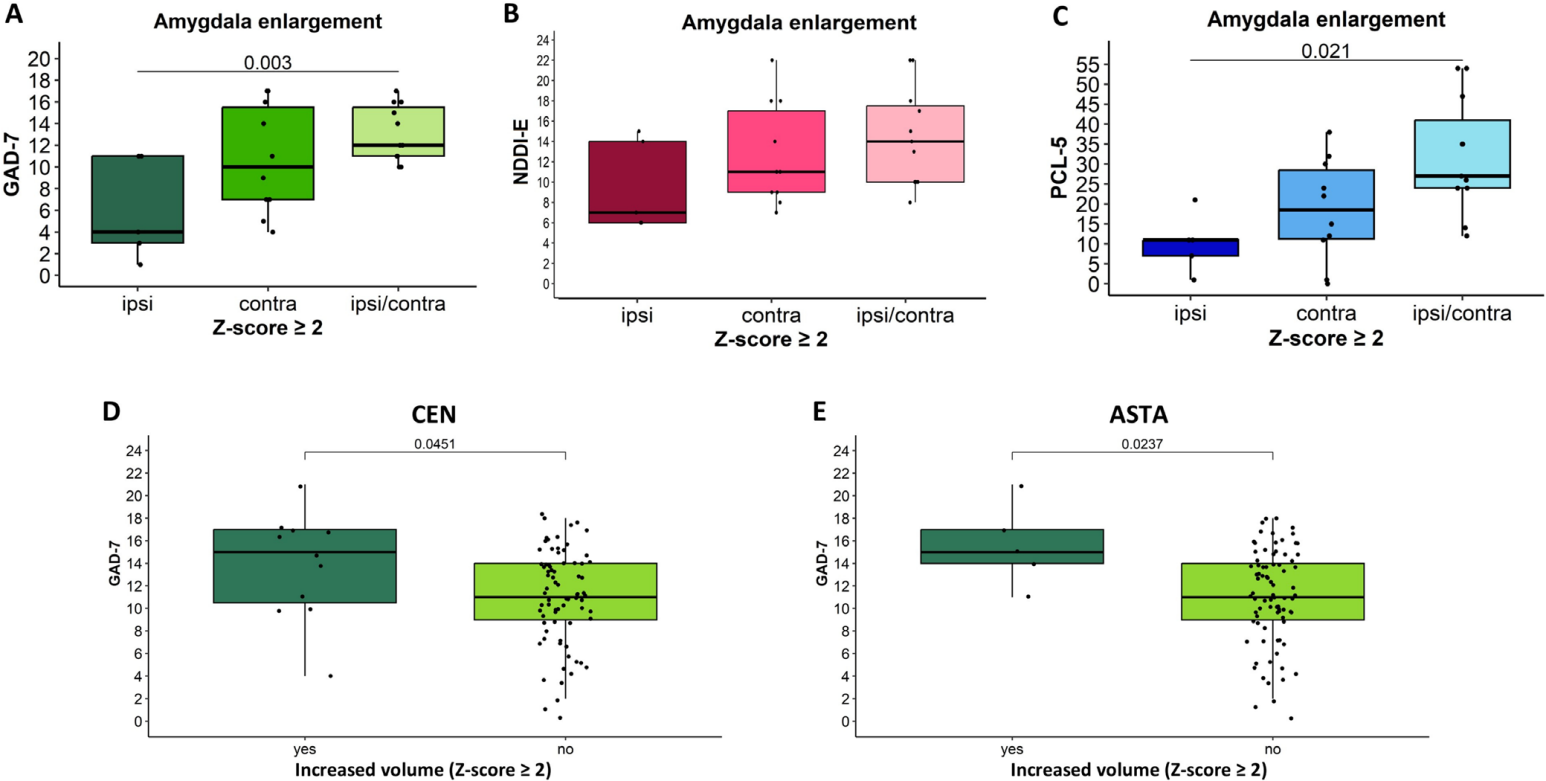
Psychiatric scores depending on the side of amygdala enlargement or the presence of enlarged vs non enlarged nuclei. There were more severe anxiety (A, GAD‐7) and PTSD (C, PCL‐5) symptoms in the presence of bilaterally compared to ipsilaterally significantly enlarged (*z*‐score ≥ 2.0) amygdala (ANOVA). The same trend was observed for depression (B, NDDI‐E), however not reaching statistical significance. Central nucleus (D) and amygdalo‐striatal transition area (ASTA, E) enlargement were associated with significantly higher anxiety (GAD‐7) scores compared to the patients without enlargement of these nuclei.

The symptoms of anxiety (higher GAD‐7 scores, *p* = 0.003) and PTSD (higher PCL‐5 scores, *p* = 0.021, ANOVA) were more severe in patients with significant bilateral AE (*z*‐score ≥ 2.0) compared to those with ipsilateral AE. The same trend was observed for NDDI‐E scores; however, it did not reach statistical significance (Figure 5A–C). The above‐described differences were not significant if patients with moderate AE were included (*z*‐score ≥ 1.5).

#### 3.4.2 Nuclei Volumes and Psychiatric Scores

In the overall cohort, no significant correlations were found between the nuclei volume *z*‐scores and the psychiatric scores. However, a higher proportion of patients with CEN enlargement (*z*‐score ≥ 1.5) screened positive for a high probability of depression compared to those without (*p* = 0.025, Chi‐Square). Furthermore, higher anxiety (GAD‐7) scores were observed in patients with significant enlargement (*z*‐score ≥ 2.0) of the CEN (*p* = 0.045) or ASTA (*p* = 0.024, t‐test) nuclei compared to those without such enlargement (Figure 5D,E). No significant differences were observed for the remaining nuclei between patients with and without enlargement of the respective nuclei. No correlations between the volume of the different nuclei and psychiatric scores were found for patients without nuclei enlargement.

Among patients with nuclei enlargement (if *n* ≥ 6 per group), we analyzed the same correlations depending on the enlargement location according to epilepsy side (ipsilateral, contralateral or ipsi/contralateral), lateralization (left or right) and epilepsy type (temporal or extratemporal). The results for each comorbidity are summarized in Figure S1. No significant correlations were found, except for a negative correlation between ATA volume and depression score in patients with bilateral epilepsy.

**FIGURE 6:**
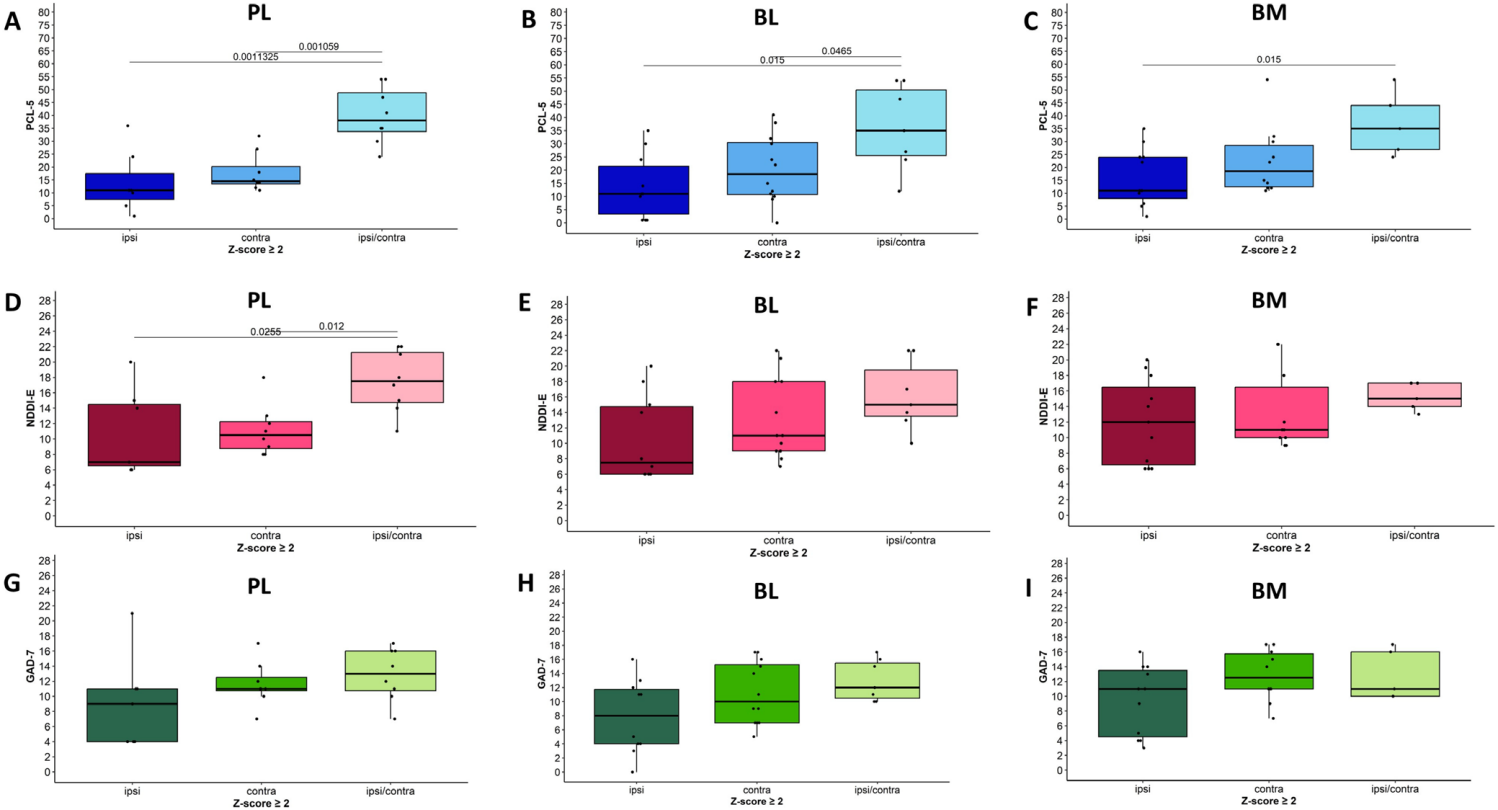
Psychiatric scores in patients with ipsilateral versus contralateral or bilateral enlargement of amygdala nuclei with respect to the epilepsy side. There were more severe PTSD (A–C) symptoms in the presence of bilateral compared to ipsilateral, significant enlargement (*z*‐scores ≥ 2.0) of the paralaminar (PL), basolateral (BL), or accessory basal (BM) nuclei (ANOVA). Depression symptoms (D–F) were more severe in presence of bilateral versus ipsi‐ or contralateral PL enlargement (D), with the same trend for the BL, not reaching statistical significance (E), and no difference for the BM (F). No statistically significant link with the side of PL, BL or BM nuclei enlargement was present for the anxiety scores (G–I).

Finally, we compared mean psychiatric scores for anxiety, depression, and PTSD between groups with ipsilateral, contralateral, and bilateral (ipsi/contra) nuclei enlargement. Statistically significant results are summarized in Figure 6. PTSD symptoms were more severe (higher PCL‐5 scores) in patients with bilateral compared to ipsilateral significant enlargement (*z*‐scores ≥ 2.0) of PL (*p* < 0.001), BL (*p* = 0.015), or BM (*p* = 0.015) nuclei (ANOVA). The NDDI‐E scores were also higher in the presence of bilateral PL enlargement (*p* = 0.026) compared to ipsi‐ or contralateral enlargement (*z*‐scores ≥ 2.0), with a similar trend observed for the BL, though it did not reach statistical significance. No differences were noted for the BM. No significant association was found between the side of nuclei enlargement and anxiety scores, nor were there significant links between comorbidity scores and the side of enlargement for any of the other nuclei studied.

### 3.5 Links Between Amygdala Volume and Other Clinical Variables

We analyzed correlations between the whole amygdala and nuclei volume *z*‐scores with clinical variables, including the age of epilepsy onset, duration of epilepsy, and seizure frequency. No correlations were found between these three variables and the whole amygdala volume. However, seizure frequency was negatively correlated with the volume of the BM and ASTA nuclei (*p* = 0.049 and *p* = 0.041, respectively, Pearson). No significant associations were found between the whole amygdala or nuclei volume and the presence of MRI‐visible lesions or etiology. Complex models controlling for confounding factors revealed no significant effects of seizure frequency, brain lesions, epilepsy type, or etiology on amygdala volume.

## 4 Discussion

The present study explored volumetric changes of the whole amygdala and its nine main nuclei using the advanced resolution of 7 T MRI and investigated their links with psychiatric comorbidities in a large cohort of 87 patients with drug‐resistant focal epilepsy.

### 4.1 Psychiatric Comorbidities

Psychiatric comorbidities were highly prevalent, and 65% of patients had a past history of at least one comorbidity. Most patients (87%) screened positive for a high probability of generalized anxiety using a GAD‐7 score cut‐off > 7, validated in French [33], which is much higher than the overall prevalence in epilepsy patients (22.9%–29%) [37] and higher than previously reported for drug‐resistant patients (30%) [28]. However, studies conducted in other languages have employed variable cut‐off scores, often excluding patients with mild anxiety symptoms when reporting the prevalence of generalized anxiety in people with epilepsy [38]. For instance, a recent German study by Hagemann et al. [39] reported that 20% of patients with epilepsy exhibited moderate to severe generalized anxiety symptoms (cut‐off > 9). However, including patients with mild symptoms (cut‐off > 4) would increase this percentage to 51%. Moreover, the percentage of patients above the GAD‐7 cut‐off in the present study aligns with the findings from a previous study by our group [40], which reported a GAD‐7 score > 7 in 67.6% of patients. These findings illustrate potential fluctuations in the severity of anxiety symptoms, suggesting that even if the prevalence of generalized anxiety disorder as a chronic condition may be lower, anxiety symptoms are highly prevalent and underdiagnosed in people with epilepsy [38].

The psychiatric diagnosis was confirmed by a structured clinical interview for all patients who were screened positive for a high probability of depression (NDDI‐E > 15) or PTSD (PCL‐5 > 31). The rates of depression (38%) were similar to those reported in a recent meta‐analysis focusing on patients undergoing epilepsy surgery [41], but twice as high as the average rates in patients with epilepsy (20%–26%) [28, 42]. PTSD was present in nearly one third of patients (28%), confirming the results of a previous study [29], while 63% of our patients had a history of traumatic exposure. Furthermore, we observed a strong positive correlation between anxiety, depression, and PTSD scores, suggesting that psychiatric comorbidities in drug‐resistant focal epilepsy often coexist and their severity can be enhanced reciprocally.

### 4.2 Volumetric Profiles of the Amygdala and Its Nuclei

Using an atlas‐based amygdala segmentation at 7 T, we identified a high prevalence of AE in the present cohort, significantly exceeding earlier estimates [2, 3, 7]. AE was present in 41% of our patients using a *z*‐score threshold of ≥ 1.5 (moderate and significant AE), and in 30% when applying a threshold of ≥ 2 (significant AE only). In comparison, a previous study reported 18.4% of AE in a population with focal epilepsy using the same ≥ 2 threshold [7]. In the subgroup of TLE patients, AE was also more frequent (37%) in our study compared to earlier publications, which reported AE rates of 12%–14% for mesial TLE [2, 3]. These differences can be due to the enhanced sensitivity of our volumetry method but may also reflect a higher AE prevalence in drug‐resistant compared to drug‐sensitive patients. Indeed, AE can ultimately regress in patients whose seizures become controlled by medication [8].

Another key finding of our study is the high prevalence of bilateral AE, accounting for 30% of all AE cases and 42% of significant AEs. The latter were predominantly contralateral or bilateral, with only 15% of cases being ipsilateral to the epilepsy side, in line with the literature [7]. The prevalence of AE was similar among patients with normal or lesional MRI and across temporal and extra‐temporal epilepsies, confirming that AE is a common abnormality in various types of focal epilepsy.

To our knowledge, this study is the first to provide volumetric characteristics of all nine main amygdala nuclei in a large cohort including both temporal and extratemporal epilepsy patients, while previous studies mostly focused on TLE [43]. Enlargement was observed across all nuclei, with the basolateral group, the AAA, and the CMN showing the highest prevalence (38%–47%). It was bilateral in about one‐third of cases. A recent study using 7 T MRI‐based FreeSurfer segmentation [43] reported enlargement only of the medial nucleus in non‐lesional temporal and extratemporal epilepsy. This discrepancy could be due to a higher sensitivity of our atlas‐based segmentation method.

Finally, we observed a low prevalence of whole amygdala atrophy (2.3%), as well as across its nuclei, ranging from 1% in the ASTA to 7% in the PL. The existing literature, primarily on temporal epilepsy, is sparse and heterogeneous. While earlier research showed reduced amygdala volumes in drug‐resistant TLE [44], more recent studies did not identify any significant amygdala volume changes [45]. In contrast, the above‐mentioned 7 T MRI study by Ballerini et al. [43] reported ipsilateral atrophy of the whole amygdala and the basolateral complex in TLE with hippocampal sclerosis. Notably, only two cases of hippocampal sclerosis were identified in our study, one of which showed an AE contralateral to the epilepsy side.

### 4.3 Amygdala Volumetric Changes and Psychiatric Scores

No statistically significant associations were found between the whole amygdala volumes and psychiatric comorbidities scores in the entire cohort. However, such links were identified in the subgroup of patients with bilateral AE, suggesting rather a pathophysiological relationship between AE and psychiatric comorbidities than a link between AE and epilepsy itself. In this subgroup, both PTSD and depression scores positively correlated with amygdala volume *z*‐scores indicating that greater AE is associated with more severe depression and PTSD symptoms. For depression, this finding is in agreement with our pilot study [10] and with a recent study in bipolar spectrum disorder (BSD), which showed greater bilateral amygdala volume in individuals with BSD compared to low‐risk individuals [46]. Furthermore, in our study, PTSD and anxiety scores were significantly higher in patients with bilateral AE than in those with ipsi‐ or contralateral AE. Taken together, these results suggest that bilateral AE may serve as a marker of psychopathogenesis in drug‐resistant focal epilepsy, particularly indicative of the severity of co‐existent PTSD and depression symptoms. Metabolic imaging data further support the role of the anterior temporal structures, including the amygdala, hippocampus, uncus, and temporal pole, in psychopathogenesis in TLE [31, 47].

### 4.4 Nuclei Enlargement and Psychiatric Scores

Our study suggests that several distinct amygdala nuclei likely contribute to each psychiatric comorbidity. For anxiety, enlargement of the CEN and ASTA was associated with more severe anxiety symptoms, compared to patients without enlargement of these nuclei. The involvement of CEN is particularly relevant, as it represents an important output hub of the amygdala, projecting to the hypothalamus, orbitofrontal cortex, rhinal cortex, and the hippocampus and is involved in the processing of autonomic, hormonal, and somatic signs of fear and anxiety [17]. Accordingly, a study in patients with generalized anxiety disorder demonstrated an increased volume of the centromedial amygdala subregion, including CEN [48]. The association of ASTA enlargement with more severe anxiety symptoms remains a more exploratory finding, since its role in emotion processing is less understood. A recent 7 T functional MRI study suggested ASTA to be involved in the awareness and decision‐making related to salient stimuli, together with the basolateral and superficial amygdala subregions [49].

For depression, our study highlighted the role of the CEN and PL nuclei. CEN enlargement, regardless of its side, was associated with a higher prevalence of positive screening for a high probability of depression. Bilateral enlargement of the PL was associated with more severe depression symptoms. The literature data related to depression are heterogeneous. One study reported atrophy of the central and cortico‐medial nuclei, and enlargement of the basolateral group nuclei, including PL, in severe depression [22]. In contrast, the above‐mentioned study on BSD showed larger volumes of the left medial and central nuclei in the BSD group compared to the high‐risk group, and a larger volume of the right lateral nucleus compared to the low‐risk group [46]. The only available study in patients with epilepsy reported an increased volume of the ipsilateral lateral nucleus in MRI‐negative TLE patients with a mixed anxiety‐depressive disorder [43]. Taken together, these data support the role of CEN and the nuclei of the basolateral group as morphological markers for depressive syndrome in focal epilepsy; however, without extrapolating to depressive disorders in general.

For PTSD, bilateral enlargement of PL, BL, and BM (the basolateral group) was linked with more severe PTSD symptoms, suggesting that these specific nuclei may drive the observed whole amygdala changes. This aligns with animal models of chronic stress showing selective hypertrophy in the basolateral complex [50]. Conversely, in non‐epileptic military veterans, PTSD was associated with smaller lateral and paralaminar nuclei and larger central, medial, and cortical nuclei [26], suggesting that the pathophysiology of PTSD in drug‐resistant focal epilepsy should be different. Noteworthy, epilepsy started in childhood or adolescence in most of our patients, with a high incidence of trauma exposure. The human PL contains immature excitatory neurons supporting persistent plasticity [51], which may result in bilateral enlargement of the basolateral group nuclei and contribute to both severe PTSD or depression and enhanced epileptogenesis, as demonstrated in an animal model [30].

### 4.5 Study Limitations

Several limitations should be mentioned. One limitation of this study is the use of screening instruments (NDDI‐E, GAD‐7, and PCL‐5) to assess psychiatric comorbidities rather than structured clinical interviews, which are the gold standard for diagnosing psychiatric disorders. The GAD‐7, in particular, is a state‐dependent measure that assesses anxiety symptoms experienced over the preceding 2 weeks rather than a trait‐dependent measure that reflects a long‐term diagnosis of generalized anxiety disorder. Consequently, the prevalence of anxiety symptoms reported in this study may be influenced by short‐term fluctuations rather than representing chronic anxiety. Despite these limitations, the strong correlations observed between different psychiatric scales (e.g., NDDI‐E and GAD‐7, PCL‐5 and NDDI‐E) and the consistency of our findings with prior literature suggest that the screening tools provided a meaningful, albeit preliminary, assessment of psychiatric comorbidities in drug‐resistant focal epilepsy.

Another limitation of this study is the variability in the time interval between the 7 T MRI scans and psychiatric assessments, ranging from several weeks to two and a half years. This temporal lag introduces the possibility that changes in psychiatric symptoms may have occurred between the imaging and screening assessments, potentially affecting the observed associations between amygdala volumetry and psychiatric comorbidities. While psychiatric disorders such as anxiety, depression, and PTSD are typically chronic conditions, and 65% of patients had a well‐documented history of psychiatric comorbidities, their severity can fluctuate over time, particularly in individuals with epilepsy. Due to the study design, we could not control for this variability or assess whether symptom stability influenced our findings. However, given that the prevalence rates of psychiatric comorbidities in our cohort align with previous studies and that significant correlations were found between specific amygdala nuclei and psychiatric scores, we believe that the overall conclusions remain robust. Future studies systematically incorporating structured psychiatric interviews and longitudinal assessments would be valuable to confirm these associations and provide a more comprehensive understanding of the relationship between amygdala enlargement and psychiatric symptoms in epilepsy.

## 5 Conclusion

In drug‐resistant focal epilepsy, psychiatric comorbidities, including anxiety, depression, and post‐traumatic stress disorder, are highly prevalent. Amygdala enlargement is frequent (40%) in this context, bilateral in 30% of cases, and is observed across all nuclei, while atrophy is rare. Bilateral whole‐amygdala enlargement correlated with the severity of co‐existing PTSD and depression symptoms. Enlargement of the central nucleus was associated with a higher prevalence of positive screening for depression and more severe anxiety symptoms. Bilateral enlargement of the paralaminar nucleus was linked to more severe depression symptoms, and bilateral enlargement of the paralaminar, basolateral, and accessory basal nuclei to more severe PTSD symptoms. These findings suggest bilateral AE as a potential marker of psychiatric comorbidities in epilepsy, warranting further research into the specific roles of amygdala nuclei in psycho‐epileptogenesis.

## Author Contributions

**Hélène Mourre:** conception and design of the study, acquisition and analysis of data, drafting the manuscript and figures. **Julia Makhalova:** conception and design of the study, acquisition and analysis of data, drafting the manuscript and figures. **Lisa Soncin:** acquisition and analysis of data, drafting the manuscript. **Elodie Garnier:** acquisition and analysis of data, drafting the manuscript and figures. **Hugo Dary:** acquisition and analysis of data, drafting the manuscript and figures. **Arnaud Le Troter:** acquisition and analysis of data. **Roy A. M. Haast:** analysis of data, drafting the manuscript and figures. **Benoit Testud:** acquisition and analysis of data. **Marie Arthuis:** acquisition and analysis of data. **Samuel Medina Villalon:** analysis of data, drafting the manuscript. **Stanislas Lagarde:** acquisition and analysis of data, drafting the manuscript. **Francesca Pizzo:** acquisition and analysis of data, drafting the manuscript. **Christian Bénar:** data analysis, drafting the manuscript. **Jean‐Philippe Ranjeva:** data analysis, drafting the manuscript. **Maxime Guye:** conception and design of the study, data analysis, drafting the manuscript. **Fabrice Bartolomei:** conception and design of the study, acquisition and analysis of data, drafting the manuscript and figures.

## Conflicts of Interest

The authors declare no conflicts of interest.

## Supporting information


**Figure S1:** Heatmap of correlation between the amygdala or nuclei volume and psychiatric scores depending on AE (*z*‐score > 1.5) location, epilepsy side and type for anxiety (A), depression (B), and PTSD (C).

## Data Availability

The data are not publicly available due to sensitive information that could compromise the privacy of research participants. Anonymized data not published within this article are available from the corresponding author upon reasonable request.
