# Supplementary material for: Nuclei‐Specific Amygdala Enlargement Is Linked to Psychiatric Comorbidities in Drug‐Resistant Focal Epilepsy
Source: Ann Clin Transl Neurol. 2025 May 19;12(7):1395–407. doi: 10.1002/acn3.70071 (PMC12257133; doi:10.1002/acn3.70071)

**Supplementary material**

**Volumetric analysis of the amygdala and its nuclei on 7T MRI**

The volumetry pipeline is illustrated in Fig. 2. MRI data were converted to the Brain Imaging Data Standard for further processing^1^. Automated volumetric segmentation of the amygdala and its nuclei was performed in all patients and HCs, combining the segmentation of the whole amygdala based on the in-house developed 7TAMI atlas^2^ with the segmentation of the amygdala nuclei based on the atlas by Tyszka and Pauli^3^ on the 7TAMI template^4^. Firstly, the Tyszka and Pauli template was registered to the high-resolution 7TAMI template and the transformation was applied to the Tyszka and Pauli atlas. Removal of the cranial band and skin signal was then performed with HD-bet^5^ on the 7TAMI template for better registration with the individual subjects. For all subjects, pre-processing steps were performed on a 3D T1-weighted magnetization-prepared 2 rapid acquisition gradient echoes sequence (MP2RAGE) to prepare the data, including correction for gradient distortion, B1^+6^ inhomogeneities, and the creation of a ‘UniDen’ (T1weighted) image, i.e. the denoised UNI image from the first and second inversion time MP2RAGE images. Segmentation was obtained by non-linear registration of the 7TAMI model with the skull-stripped (using HD-bet) UniDen image that was then applied to the whole amygdala segmentation (7TAMI) and the nuclei atlas (Tyszka and Pauli). Nine nuclei were segmented for each amygdala: Lateral nucleus (LA); Basolateral nucleus (BL); Accessory basal nucleus (BM); Paralaminar nucleus (PL); Corticomedial nucleus (CMN); Periamygdaloid cortex (ATA); Anterior amygdaloid area (AAA); Amygdalo-striatal transition area (ASTA); Central nucleus (CEN).

Finally, all volumetric measurements were extracted from left and right amygdala segmentation masks adapted to the subject's anatomy for each patient and healthy control. The left and right amygdala volumes were normalized by the total intracranial volume, extracted using FreeSurfer and the skull-stripped (performed using PreSurfer^7^) UniDen image as input, to correct for individual differences in brain volume. The z-scores of normalized amygdala volumes were then calculated for each patient compared with the volumetry of control subjects for the whole amygdala and the nine nuclei (z-score = (patient's individual amygdala volume - group's average amygdala volume of control group)/standard deviation of amygdala volume of control group). For the purposes of this study, the presence of amygdala enlargement was defined by a z-score≥1.5 to include cases with a moderate enlargement (1.5≤zscore<2)^2,8^. Significant enlargement was determined by a z-score≥2. Statistical analyses were performed for both thresholds (z≥1.5 and z≥2).

**References**

1. Gorgolewski KJ, Auer T, Calhoun VD, et al. The brain imaging data structure, a format for organizing and describing outputs of neuroimaging experiments [Internet]. Sci. data 2016;3[cited 2024 Nov 17 ] Available from: https://pubmed.ncbi.nlm.nih.gov/27326542/

2. Makhalova J, Le Troter A, Aubert-Conil S, et al. Epileptogenic networks in drug-resistant epilepsy with amygdala enlargement: Assessment with stereo-EEG and 7 T MRI [Internet]. Clin. Neurophysiol. 2021;133:94–103.[cited 2021 Dec 10 ] Available from: https://pubmed.ncbi.nlm.nih.gov/34826646/

3. Tyszka JM, Pauli WM. In vivo delineation of subdivisions of the human amygdaloid complex in a high-resolution group template [Internet]. Hum. Brain Mapp. 2016;37(11):3979–3998.[cited 2020 Jun 30 ] Available from: /pmc/articles/PMC5087325/?report=abstract

4. Brun G, Testud B, Girard OM, et al. Automatic segmentation of deep grey nuclei using a high-resolution 7T magnetic resonance imaging atlas-Quantification of T1 values in healthy volunteers [Internet]. Eur. J. Neurosci. 2022;55(2):438–460.[cited 2022 Sep 13 ] Available from: https://pubmed.ncbi.nlm.nih.gov/34939245/

5. Isensee F, Schell M, Pflueger I, et al. Automated brain extraction of multisequence MRI using artificial neural networks [Internet]. Hum. Brain Mapp. 2019;40(17):4952–4964.[cited 2024 Aug 20 ] Available from: https://pubmed.ncbi.nlm.nih.gov/31403237/

6. Massire A, Seiler C, Troalen T, et al. T1-Based Synthetic Magnetic Resonance Contrasts Improve Multiple Sclerosis and Focal Epilepsy Imaging at 7 T [Internet]. Invest. Radiol. 2021;56(2):127–133.[cited 2024 Aug 20 ] Available from: https://pubmed.ncbi.nlm.nih.gov/32852445/

7. Kashyap S. srikash/presurfer: ondu [Internet]. [date unknown];[cited 2024 Nov 17 ] Available from: https://zenodo.org/records/4626841

8. Coan AC, Morita ME, Campos BM, et al. Amygdala enlargement occurs in patients with mesial temporal lobe epilepsy and hippocampal sclerosis with early epilepsy onset. Epilepsy Behav 2013;29(2):390–394.

**Supplementary Figure 1. Heatmap of correlation between the amygdala or nuclei volume and psychiatric scores depending on AE (z-score >1.5) location, epilepsy side and type for anxiety (A), depression (B), and PTSD (C).**

*statistically significant. The correlations for the enlargement of distinct nuclei are shown only if n≥6 subjects per respective group. The whole amygdala volume positively correlated with depression and PTSD scores when bilateral (ipsi/contralateral) amygdala enlargement was present. No significant correlations were found for different nuclei, except for a negative correlation between the volume of the peramygdaloid cortex (ATA) and depression score in patients with bilateral epilepsy. Abbreviations: AE, amygdala enlargement; AMY, amygdala; La, Lateral nucleus; BL, Basolateral nucleus; BM, Accessory basal nucleus; CEN, Central nucleus; CMB, Corticomedial nucleus; PL, Paralaminar nucleus; ATA, Periamygdaloid cortex; ASTA, Amygdalo-striatal transition area; AAA, Anterior amygdaloid area.


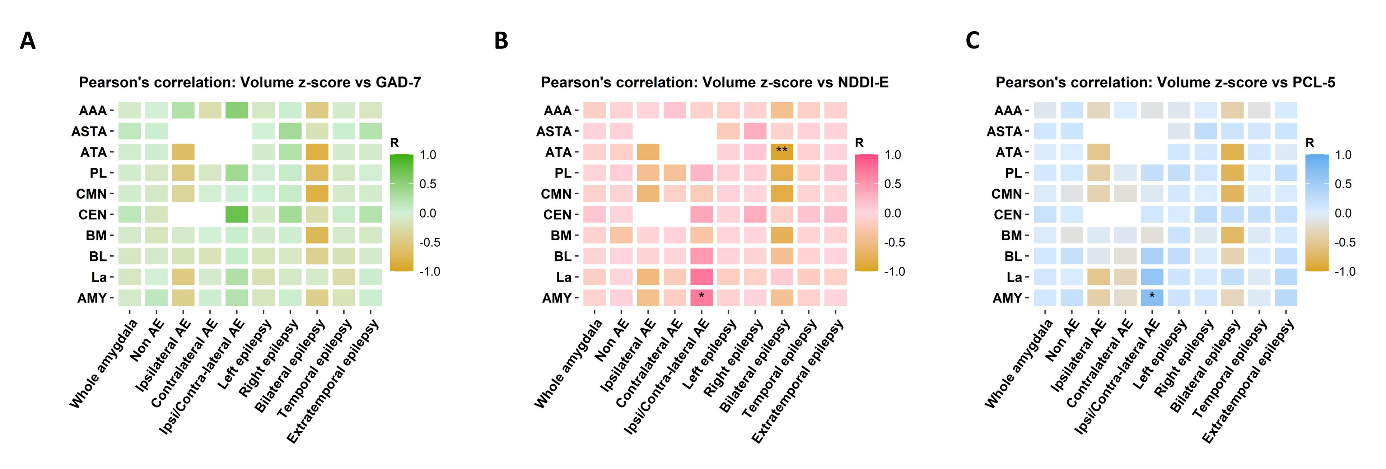

Supplement: Supplementary file 1 — Figure S1: Heatmap of correlation between the amygdala or nuclei volume and psychiatric scores depending on AE (z‐score > 1.5) location, epilepsy side and type for anxiety (A), depression (B), and PTSD (C). [file ACN3-12-1395-s001.docx]
